# Effects of probiotics on the oral health of patients undergoing orthodontic treatment: a systematic review and meta-analysis

**DOI:** 10.1093/ejo/cjad046

**Published:** 2023-08-08

**Authors:** Wener Chen, Jianhan Ren, Jiachen Li, Simin Peng, Chengfei Zhang, Yifan Lin

**Affiliations:** Division of Paediatric Dentistry and Orthodontics, Faculty of Dentistry, The University of Hong Kong, Prince Philip Dental Hospital, 34 Hospital Road, Hong Kong SAR, China; Division of Paediatric Dentistry and Orthodontics, Faculty of Dentistry, The University of Hong Kong, Prince Philip Dental Hospital, 34 Hospital Road, Hong Kong SAR, China; Division of Paediatric Dentistry and Orthodontics, Faculty of Dentistry, The University of Hong Kong, Prince Philip Dental Hospital, 34 Hospital Road, Hong Kong SAR, China; Division of Paediatric Dentistry and Orthodontics, Faculty of Dentistry, The University of Hong Kong, Prince Philip Dental Hospital, 34 Hospital Road, Hong Kong SAR, China; Division of Restorative Dental Sciences, Faculty of Dentistry, The University of Hong Kong, Prince Philip Dental Hospital, 34 Hospital Road, Hong Kong SAR, China; Division of Paediatric Dentistry and Orthodontics, Faculty of Dentistry, The University of Hong Kong, Prince Philip Dental Hospital, 34 Hospital Road, Hong Kong SAR, China

**Keywords:** probiotics, orthodontic treatment, oral health, oral microorganisms, meta-analysis

## Abstract

**Background and objective:**

The effect of probiotics on oral health maintenance in orthodontic patients remains controversial. The aim of the study is to systematically review and assess the effects of probiotics on the oral health and microbiome of patients undergoing orthodontic treatment.

**Search methods and selection criteria:**

Databases including PubMed, Web of Science, Cochrane Library, ClinicalTrials.gov, and ProQuest Dissertations & Theses Global databases were searched from their inception until June 2022. Randomised controlled trials that assessed the effects of probiotics on clinical and microbial outcomes in patients undergoing orthodontic treatment were included.

**Data collection and analysis:**

Data screening and collection were performed, and the risk of bias (RoB) was assessed using the Cochrane RoB 2 tool. The meta-analysis evaluated the effects of probiotics on *Streptococcus mutans* (*S. mutans*) and *Lactobacillus* counts. The quality of the evidence from the meta-analyses was assessed with Grading of Recommendations Assessment, Development and Evaluation (GRADE).

**Results:**

A total of 405 records were identified, of which 15 studies were included in the qualitative synthesis and 4 in the meta-analysis. The patients in all the included studies were treated with fixed orthodontic appliances. Results regarding clinical outcomes were controversial; four out of five studies reported no significant changes in plaque in the probiotic group (*P* > .05), and two out of three studies reported no significant changes in the gingival index (*P* > .05). Regarding microbial outcomes, the meta-analysis results revealed that probiotics significantly increased the likelihood of reducing the abundance of *S. mutans* to below 10^5^ CFU/ml (risk ratio: 2.05 [1.54, 2.72], *P* < .001) and reduced the likelihood of increasing the abundance of *S. mutans* to beyond 10^6^ CFU/ml (risk ratio: 0.48 [0.28, 0.83], *P* = .009). However, the quality of evidence according to the GRADE was moderate.

**Conclusions and implications:**

There is insufficient evidence to determine the clinical benefits of probiotics as a supplement for the oral health of patients undergoing orthodontic treatment. However, probiotics may have benefits in reducing the salivary *S. mutans* counts in orthodontic patients.

**Registration:**

PROSPERO (CRD42022366650).

## Introduction

Orthodontic treatment aims to correct occlusal anomalies and enhance facial aesthetics. However, the use of brackets, elastics, and archwires in fixed orthodontic treatment or the full coverage of teeth by clear aligners pose a significant challenge to maintaining oral hygiene. These orthodontic tools create retentive areas on the tooth surface, favourable for microorganisms and food accumulation. If not timely removed, enamel demineralisation and gingivitis would be developed [1, 2]. Furthermore, multiple studies have demonstrated that patients undergoing fixed orthodontic treatment are susceptible to gingival inflammation and enamel demineralisation [3–5]. *Streptococcus mutans* (*S. mutans*) and *Lactobacillus acidophilus* are the most common colonisers responsible for these consequences [6, 7]. Oral hygiene strategies, including fluoride application, antimicrobial oral rinses, and dietary modifications, have been proposed to prevent the hazards of orthodontic treatment on the tooth structures and gingival tissues [8, 9]. Nevertheless, patients undergoing orthodontic treatment still have a high risk of dental caries and gingivitis.

Probiotics have been proposed as a novel method for oral health maintenance. They refer to ‘live microorganisms, when administered in sufficient quantities, provide health benefits to the host’, which are available in various forms, such as lozenges, tablets, mouthwashes, and yoghurt [10]. The functions of probiotics are to modulate immunoinflammatory responses by producing bioactive substances, such as bacteriocins or organic acids, and competing with pathogenic bacteria after adhering to the oral cavity [11]. *Lactobacillus* and *Bifidobacterium* are the most commonly used probiotic genera in orthodontic treatment [12].

Studies have shown that probiotics are beneficial in preventing caries, gingivitis, and halitosis [13–15]. For example, Pahumunto *et al*. reported that the probiotic milk containing *Lactobacillus paracasei* SD1 significantly decreased the development of caries and the number of *S.mutans* in preschoolers as compared to the placebo [16]. Vicario *et al*. assessed the clinical effect of *Lactobacillus reuteri* Prodentis (GUM, Sunstar, Switzerland) in the treatment of periodontitis and found that the mean bleeding on probing and pocket probing depths significantly decreased by 26% and 4.8 mm in the probiotic group, respectively [17]. Lee *et al*. evaluated the effects of *Weissella cibaria* Chonnam Medical University (CMU) (oraCMU; OraPharm, Inc., Seoul, South Korea) on halitosis and found that the volatile sulphur compounds level significantly decreased by 4.81 in the probiotic group [18].

The effects of probiotics on patients undergoing orthodontic treatment remain controversial due to the varied results reported by multiple studies [19–22]. Two published systematic reviews have evaluated the effects of probiotics on the oral health of individuals undergoing orthodontic treatment [23, 24]. Hadj-Hamou *et al*. systematically reviewed the clinical effects of probiotics on the inflammation of the gingival tissues and the decalcification of the enamel in orthodontic patients. The review included four studies and concluded that supplementation of probiotics did not affect the development of inflammation in the gingivae or decalcification in the enamel [23]. In another review, Pietri *et al*. assessed nine studies and qualitatively concluded that probiotics had antimicrobial activity against oral pathogenic bacteria [24]. However, neither review employed quantitative syntheses (i.e. meta-analysis) to comprehensively analyse the clinical and microbial effects. Recently, a few more randomised controlled trials (RCTs) assessing the effects of probiotics on patients undergoing orthodontic treatment have emerged as the latest evidence, warranting an updated summary of all the evidence [25–27]. The current study aimed to systematically synthesise data from the available literature to assess the clinical and microbial effects of probiotics on patients undergoing orthodontic treatment. Specifically, the study analysed clinical outcomes including white spot lesions (WSLs) and periodontal indexes (gingival index [GI] and plaque index [PI]), as well as the microbial outcome of salivary bacterial counts.

## Materials and methods

### Protocol and registration

This systematic review was conducted according to the Preferred Reporting Items for Systematic Reviews and Meta-analysis (PRISMA) guidelines. The study protocol has been registered in the PROSPERO database (No.: CRD42022366650). A detailed PRISMA checklist has been appended in Supplementary Table 1.

### Eligibility criteria

The study followed the PICOS format, with the following criteria: (i) Population: healthy patients undergoing orthodontic treatment; (ii) Intervention: the use of probiotics; (iii) Comparison: placebo or alternative treatment or no intervention; (iv) Outcomes: clinical outcomes, which comprise periodontal-related indexes (GI and PI) and incidence of WSL, as well as microbial outcomes, which include bacterial counts; and (v) Study design: RCT. Thus, the overall study objective based on the PICOS format was as follows: what are the clinical and microbial effects of probiotics on the oral health of patients undergoing orthodontic treatment? The detailed inclusion and exclusion criteria are listed in Table 1.

**Table 1.** Eligibility criteria for study selection.

Category	Inclusion criteria	Exclusion criteria
Study design	Randomised controlled trials	Studies that are not randomised controlled trials
Participants	Healthy patients undergoing orthodontic treatment	Patients with craniofacial anomalies, periodontitis, and systemic or other medical conditions
Interventions	Use of probiotics	Use of non-probiotics
Comparisons	Placebo Alternative treatment No intervention	Studies without a control group
Outcomes	Clinical outcomes: periodontal indexes (GI and PI) and incidence of WSLs Microbial outcome: bacterial counts	Other outcomes

GI, gingival index; PI, plaque index; WSLs, white spot lesions.

### Information sources and search strategy

The following electronic databases were searched by two authors (JR and WC) for the relevant literature published from the inception of each database until June 2022: PubMed, Web of Science, Cochrane Library, ClinicalTrials.gov, and ProQuest Dissertations & Theses Global. Furthermore, three major orthodontic journals (*American Journal of Orthodontics and Dentofacial Orthopedics*, *Angle Orthodontist*, and *European Journal of Orthodontics*) from inception to June 2022 and the reference lists of the selected articles were also hand searched. The following search terms were used: ‘probiotic OR probiotics OR *Lactobacillus* OR *Bifidobacterium*’ AND ‘orthodontic OR orthodontics OR bracket OR brackets OR brace OR braces OR fixed appliance OR fixed appliances OR aligners OR aligner OR Invisalign’.

### Study selection and data collection

The shortlisted studies were screened based on the titles and abstracts independently by two authors (WC and JL) to identify the available studies. Full-text papers were retrieved for additional evaluation when the titles and abstracts of the papers lacked adequate information. Disagreements were resolved through discussion with a third author (YL). The Kappa coefficient of agreement between the two reviewers was 0.80 for the screening of titles and abstracts and 0.89 for the screening of full texts. Data from the selected studies were extracted into specific extraction tables, and the following terms were recorded for each study: author names and year of publication, study design, baseline participant characteristics (age, sex ratio, number of participants, and type of orthodontic treatment), study groups, probiotic microorganisms and usage, clinical and microbiological parameters, follow-up duration, and main conclusions.

### Risk-of-bias assessment

The risk of bias (RoB) was independently assessed by two authors (WC and YL) using the Cochrane RoB tool for randomised trials (RoB 2.0). The quality assessment criteria spanned five domains: randomisation process, deviations from intended interventions, missing outcome data, outcome measurement, and reported result selection. The RoB in a study was classified as ‘low’ if all the domains were judged to have a low-risk bias; ‘some concerns’ if at least one domain was judged to have some concern bias but not a high RoB for any domain; and ‘high RoB’ if at least one domain was judged to have a high RoB or included some concerns for multiple domains.

### Summary measures and quantitative synthesis of the results

Using the RevMan software (Review Manager version 5.3; Copenhagen: the Nordic Cochrane Centre, the Cochrane Collaboration, 2014), a meta-analysis of dichotomous outcomes was performed to compare the number of probiotic-treated patients with those in the control group with high (>10^6^ CFU/ml) and low (<10^5^ CFU/ml) *S. mutans* and *Lactobacillus* counts after treatment. Studies with a ‘high RoB’ were excluded from the meta-analysis. The risk ratios and 95% confidence intervals were calculated and displayed in forest plots. A *P* value < .05 indicated statistical significance. The *I*^2^ test was performed to assess the heterogeneity of the studies. The fixed effects model was applied if the *I*^2^ was < 50, whereas the random effects model was used if the *I*^2^ was ≥ 50. Data that could not be analysed quantitatively were evaluated descriptively. Furthermore, due to insufficient data and variations in the included studies, subgroup analyses, analyses for ‘small-study effects’, and assessment of publication bias could not be carried out. The quality of the evidence from the meta-analyses was assessed using the Grading of Recommendations Assessment, Development and Evaluation (GRADE) method [28].

## Results

### Study selection

**Figure 1.**
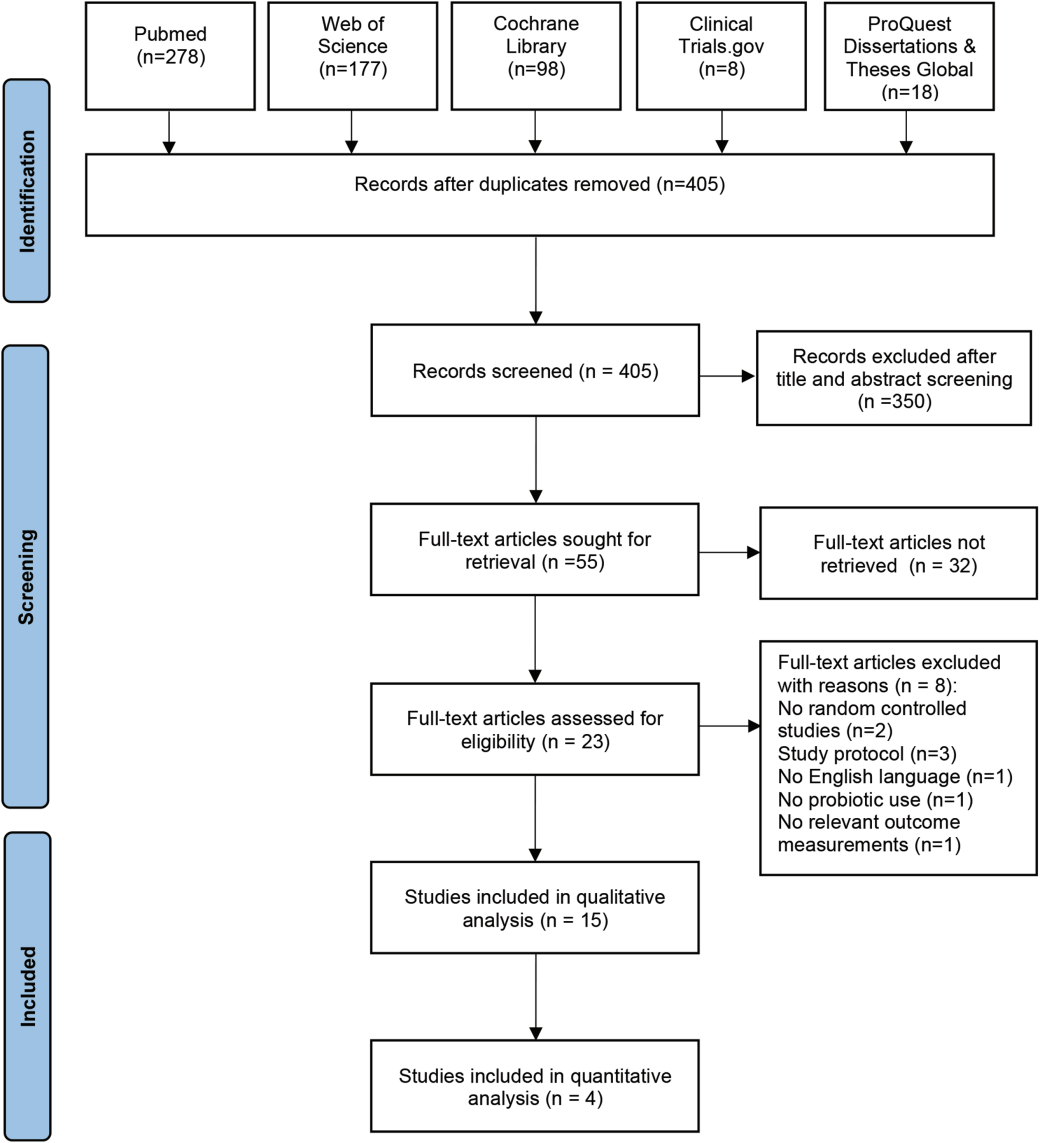
PRISMA flow diagram of the search results from the electronic databases.

The study flow is depicted in Fig. 1. The systematic search identified 579 records and 405 studies were screened after excluding duplicates. A total of 350 studies were excluded after screening for titles and abstracts. The remaining 55 studies were further evaluated through full-text screening for eligibility. Finally, a total of 15 studies were included in the qualitative synthesis (Fig. 1).

### General characteristics of the included studies

The general characteristics of the 15 studies are presented in Table 2. All of these studies were RCTs that were published between 2009 and 2022 and used a double-blind (10 RCTs), parallel-group (13 RCTs), and cross-over (2 RCTs) design. The participant age ranged between 8 and 35 years, and the sample size ranged between 24 and 85. All of the included studies had test and control groups for comparison. Although we had no restrictions in terms of the type of orthodontic appliance used, all of the included studies used fixed orthodontic appliances.

**Table 2.** Summary of the 15 studies included.

Author, year	Study design	Age range (mean)	Participants (number and gender)	Orthodontic treatment	Study groups	Study outcomes	Probiotic microorganism	Probiotic usage	Intervention duration	Additional follow-up (no intervention)	Main conclusions
Dadgar *et al*., 2021 [25]	RCT, parallel	12–20 (NR)	*N* = 38 (14M, 24F)	Fixed orthodontic treatment	Test group: probiotic mouthwash (*n* = 13) Control group: (i) placebo mouthwash (*n* = 13) (ii) sodium fluoride mouthwash (*n* = 12)	*S.mutans* counts in plaque	*L. plantarum* (10^8^CFU/30 mg)	Twice daily	14 days	/	No significant difference in *S. mutans* counts between the probiotic group and the placebo group.
Ebrahim *et al*., 2022 [26]	RCT, double-blind, parallel	11–18 (15.7 ± 1.7)	*N* = 58 (25M, 33F)	Fixed orthodontic treatment	Test group: Lorodent probiotic lozenge (*n* = 29) Control group: placebo lozenge (*n* = 29)	PI, *S.mutans* DNA levels in plaque and saliva	Probiotic complex: *S. salivarius* K12 and five probiotic strains of the genus *Lactobacillus* (3 × 10^5^CFU/lozenge)	Two lozenges two times daily for the first 7 days, followed by two lozenges once a day for the next 21 days	28 days	28 days	No significant change in PI and in *S. mutans* DNA levels in the saliva and plaque in the probiotic group during the intervention and follow-up periods.
Megha *et al*., 2019 [22]	RCT, double-blind, parallel	8–15 (NR)	*N* = 27 (14M, 13F)	Fixed orthodontic treatment	Test group: (i) probiotic yoghurt (*n* = 9) (ii) Indian curd with probiotic bacteria (*n* = 9) Control group: placebo yoghurt (*n* = 9)	*S.mutans* scores in plaque and saliva	(i) *L. acidophilus* 20 × 10^7^ CFU/g, *Bifidobacteria* 5.4 × 10^7^CFU/g-yoghurt (ii) *L.acidophilus* × 10^6^CFU/g, *S.thermophiles* 35 × 10^4^CFU/g-Indian curd	200 g once daily	14 days	/	A significant reduction in salivary *S. mutans* levels was recorded after probiotic yoghurt ingestion.
Shah *et al*., 2019 [21]	RCT, parallel	NR	*N* = 30 (M:F) NR	NR	Test group: probiotic mouthwash (*n* = 10) Control group: (i) 0.2% chlorhexidine mouthwash (*n* = 10) (ii) no intervention (*n* = 10)	PI, GI, *S.mutans* counts in saliva	*L. sporogenes* (2 × 10^8^ CFU/g)	Twice daily	28 days	/	The probiotic group had significantly reduced PI, GI, and *S.mutans* counts as compared to the control group without intervention.
Cildir *et al*., 2009 [36]	RCT, double-blind, crossover	12–16 (14 ± 1.2)	*N* = 24 (8M, 16F)	Fixed orthodontic treatment	Test group: probiotic yoghurt (*n* = 12) Control group: placebo yoghurt (*n* = 12)	*S. mutans* and *Lactobacillus* scores in saliva	*B. animalis subsp. Lactis* DN-173010 (2 × 10^8^ CFU/g)	200g once daily	T1. 7 days (run-in) T2.14 days (intervention) T3. 42 days (wash-out) T4.14 days (intervention)	/	A statistically significant reduction of salivary *S.mutans* in the probiotic group. No significant alterations of the salivary *Lactobacilus* counts were observed.
Pinto *et al*., 2014 [33]	RCT, double-blind, crossover	10–30 (15)	*N* = 26 (10M, 16F) (overall 30, 4 were excluded due to non-attendance)	Fixed orthodontic treatment	Test group: probiotic yoghurt (*n* = 15) Control group: placebo yoghurt (*n* = 15)	Total cultivable microorganisms counts, *S. mutans* and *Lactobacilli* counts in plaque and saliva	*B. animalis subsp. Lactis *DN-173010	200g once daily	T1. 7 days (run-in) T2.14 days (intervention) T3. 28 days (wash-out) T4.14 days (intervention)	/	No difference between the yogurt containing probiotic and the control yogurt for any of the studied variables.
Gizani *et al*., 2016 [32]	RCT, double-blind, parallel	NR (15.9 ± 3.9)	*N* = 85 (29M, 56F)	Fixed orthodontic treatment	Test group: probiotic lozenge (*n* = 42) Control group: placebo lozenge (*n* = 43)	WSL, *S. mutans *and *Lactobacilus* scores in saliva	Two strains of the probiotic bacterium *L. reuteri* (DSM 17938 and ATCC PTA 5289) (10^8^ live bacteria of each strain)	One lozenge once daily	17 ± 6.8 months	/	No differences in the incidence of WSL and *S.mutans* counts between the groups at debonding. The levels of salivary* Lactobacillus* were significantly reduced in both groups.
Kohar *et al*., 2015 [29]	RCT, parallel	18–25 (NR)	*N* = 30 (M:F) NR	Fixed orthodontics treatment	Test group: (i) probiotic lozenge (*n* = 10) (ii) probiotic drinks (*n* = 10) Control group: placebo (*n* = 10)	PI	(i) *L. reuteri* (200 million live bacteria/lozenge)-lozenge (ii) *L. casei* strain *Shirota* (6.5 million viable cells of LcS/bottle)-drink	One lozenge/bottle once daily	14 days	/	No significant differences in the probiotic group for any variables.
Alp and Baka, 2018 [34]	RCT, parallel	12–17 (14.43 ± 1.93)	*N* = 45 (18M, 27F)	Fixed orthodontic treatment	Test group: (i) probiotic kefir (*n* = 15) (ii) probiotic toothpaste (*n* = 15) Control group: no probiotic treatment (*n* = 15)	*S. mutans* and *Lactobacillus* levels in saliva	(i) mixture of lactic acid bacteria culture-kefir (ii) Bacteriocin extracted from lactic acid bacteria-toothpaste	(i) 2 × 100 ml kefir once daily (ii) Twice daily (toothpaste)	42 days	/	A statistically significant decrease was observed in the salivary *S mutans* and *Lactobacillus* levels in the probiotic groups.
Goyal *et al*., 2019 [19]	RCT, parallel	15–35 (NR)	*N* = 30 (M:F) NR	Fixed orthodontic treatment	Test group: probiotic mouthwash (*n* = 10) Control group: (i) amine fluoride mouthwash (*n* = 10) (ii) no intervention (*n* = 10)	*P.gingivalis* levels in subgingival plaque	*L. reuteri* (0.1 billion CFU), *L. rhamnosus* (0.1 billion CFU), *B.longum* (0.06 billion CFU), *B. bifidum* (0.1 billion CFU) per gram	Twice daily	6 months	/	The levels of *P. gingivalis* were significantly decreased with probiotic mouthwash.
Benic *et al*., 2019 [20]	RCT, triple-blind, parallel	10–30 (14.9 ± 3.2)	*N* = 64 (23M, 41F)	Fixed orthodontic treatment	Test group: probiotic lozenge (*n* = 32) Control group: placebo lozenge (*n* = 32)	PI and GI	*S. salivarius* M18 (3.6 × 10^9^ CFU/lozenge)	One lozenge twice daily	1 month	3 months	PI and GI scores were not significantly influenced by the probiotic use during the intervention and follow-up periods.
Alforaidi *et al*., 2021 [27]	RCT, double-blind, parallel	NR (17.3 ± 1.1)	*N*= 28 (14M, 14F)	Fixed orthodontic treatment	Test group: probiotic mouth rinse (*n* = 14) Control group: placebo mouth rinse (*n* = 14)	*S. mutans* and *Lactobacillus* counts in saliva	*L. reuteri* DSM 17938(>1 × 10^8^CFU/5 drops), *L. reuteri* ATCC PTA 5289(>1 × 10^8^ CFU/5 drops)	Twice daily	21 days	/	No significant differences in *S. mutans* and *Lactobacillus* counts were found.
Jose *et al*., 2013 [35]	RCT, double-blind, parallel	14–29 (20)	*N* = 60 (18M, 42F)	Fixed orthodontic treatment	Test group: (i) probiotic curd (*n* = 20) (ii) probiotic toothpaste (*n* = 20) Control group: no probiotic treatment (*n* = 20)	*S. mutans* levels in plaque	(i) *L. acidophilus*-SD 5221 (10^9^ CFU/200g)- curd (ii) Bacteriocin extracted from lactic acid bacteria-toothpaste	(i) 200 mg curd once daily (ii) Twice daily (toothpaste)	30 days	/	The probiotic groups caused a significant decrease in the *S.mutans* levels in the plaque.
Habib, 2016 [31]	RCT, double-blind, parallel	11–18 (15.69 ± 1.70)	*N* = 58 (25M, 33F)	Fixed orthodontic treatment	Test group: probiotic lozenge (*n* = 29) Control group: placebo lozenge (*n* = 29)	Modified GI, periodontal pathogens levels in subgingival plaque	*S. salivarius* K12 and five probiotic strains of the genus *Lactobacillus* (1 billion CFU/lozenge)	Two lozenges two times daily for the first 7 days, followed by two lozenges once a day for the next 21 days	28 days	28 days	The probiotic lozenge did not significantly reduce periodontal pathogens or GI during the intervention and follow-up periods.
Jivraj, 2015 [30]	RCT, double-blind, parallel	11–18 (15.69 ± 1.70)	*N* = 58 (25M, 33F)	Fixed orthodontic treatment	Test group: probiotic lozenge (*n* = 29) Control group: placebo lozenge (*n* = 29)	PI, *S. mutans* and *Lactobacillus* levels in saliva and subgingival plaque	*S. salivarius* K12 and five probiotic strains of the genus *Lactobacillus* (1 billion CFU/lozenge)	Two lozenges two times daily for the first 7 days, followed by two lozenges once a day for the next 21 days	28 days	28 days	No significant changes in *S. mutans* and *Lactobacillus* levels in the probiotic group during the intervention period and follow-up period.

RCT, randomised controlled trial; NR, not reported; M, male; F, female; PI, plaque index; GI, gingival index; WSL, white spot lesion; *S., Streptococcus; L., Lactobacillus; B., Bifidobacterium; P., Porphyromona.*

The PI was recorded in five studies [20, 21, 26, 29, 30]. The GI or modified GI was recorded in three studies [20, 21, 31]. The incidence of WSL was assessed in one study [32]. The *S. mutans* and/or *Lactobacillus* counts were evaluated in the plaque or saliva of patients in 11 studies [21, 22, 25–27, 30, 32–36].

### Risk-of-bias assessment

**Figure 2.**
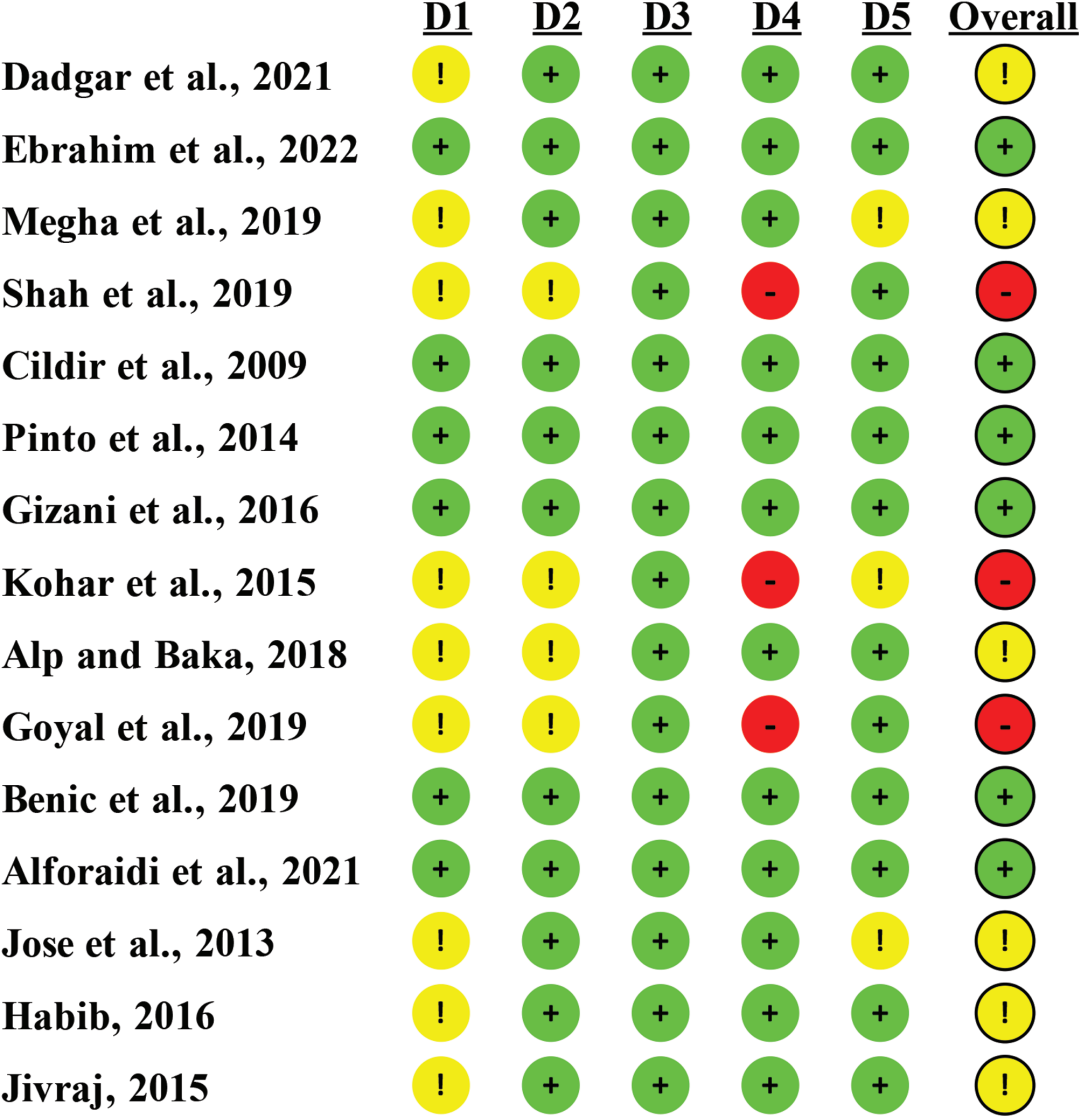
Quality assessment of the RCTs (RoB 2.0). Symbol: green (+): low RoB; yellow (!): some concerns of bias; red (−): high RoB. Domains: D1: Bias arising from the randomisation process; D2: Bias due to deviations from intended intervention; D3: Bias due to missing outcome data; D4: Bias in measurement of the outcome; D5: Bias in selection of the reported result.

The results of the RoB 2.0 assessment of the selected studies are presented in Fig. 2. Six studies were considered as ‘low RoB’ [20, 26, 27, 32, 33, 36] for all the key domains. Six studies were considered to have ‘some concerns’ [22, 25, 30, 31, 34, 35], whereas three studies [19, 21, 29] were considered to have a ‘high RoB’.

### Characteristics related to probiotic administration

The durations of the probiotic interventions ranged from 14 days to 17 ± 6.8 months, and the most typical intervention period was 14 days [22, 25, 29, 33, 36]. The measurements were taken at the beginning before probiotic administration and immediately after administration. Four studies examined the effects of probiotic treatment by measuring participants both before and after treatment, as well as at follow-up periods ranging from 28 days to 3 months after treatment cessation [20, 26, 30, 31].

Regarding probiotic delivery vehicles, four studies used mouthwash [19, 21, 25, 27], six studies used lozenges [20, 26, 29–32], three studies used yoghurt [22, 33, 36], and two studies used toothpaste [34, 35]. In terms of probiotic species, most studies used a mix of probiotic species, whereas five studies used a single probiotic species [20, 21, 25, 33, 36]. At the genus level, *Lactobacillus* species were most commonly used [19, 21, 22, 25–27, 29–32], followed by *Streptococcus* [20, 22, 26, 30, 31].

## Main outcomes of the included studies

### Clinical changes

The clinical outcome parameters used to assess the effects of probiotics on the oral health of the patients undergoing orthodontic treatment were PI, GI, and incidence of WSLs. Due to the high level of heterogeneity in the clinical examinations and parameters used, quantitative synthesis of the clinical changes could not be conducted.

Of the five studies that recorded the PI, four reported no significant changes between the probiotic and placebo groups (*P* > .05) [20, 26, 29, 30]. In contrast, Shah *et al*. [21] reported a considerably decreased PI in the probiotic group compared to the control group without intervention (*P* < .05). Specifically, the mean PI decrease was 0.6 in the probiotic group, which was considerably greater than the 0.03 decrease in the control group. In terms of the GI, Benic *et al*. and Habib [20, 31] found that probiotics did not affect the GI significantly (*P* > .05); however, Shah *et al*. [21] reported a significant reduction of 0.87 in GI of the probiotic group (*P* < .05). In terms of the incidence of WSLs, Gizani *et al*. [32] found no significant differences between the probiotic and placebo groups. At debonding, no new lesion was found in 22 out of 42 patients in the probiotic group and 26 out of 43 in the placebo group.

### Microbial changes

Of the 11 studies that assessed *S. mutans* counts in the saliva or plaque of patients, five [21, 22, 34–36] revealed that probiotic use can significantly reduce *S. mutans* counts (*P* < .05). For instance, Cildir *et al*. [36] demonstrated that the percentage of subjects with high *S. mutans* counts dropped from 63% to 21% after 14 days consumption of probiotic yoghurt. Similarly, Shah *et al*. [21] observed a significant decrease in *S. mutans* counts from 7.1 × 10^4^ CFU/ml to 1.1 × 10^3^ CFU/ml after 28 days of using probiotic mouthwash. Of the six studies [27, 30, 32–34, 36] that evaluated *Lactobacillus* counts in the saliva or plaque of patients, Alp and Baka [34] and Gizani *et al*. (32) found significant reductions in *Lactobacillus* counts (*P* < .05). Specifically, Alp and Baka [34] reported that the percentage of subjects with a *Lactobacillus* level of 10^6^ or greater at the beginning of the study decreased from 13.3% to 0% in the probiotic toothpaste group. In the study by Gizani *et al*., [32] such proportion decreased from 40.5% to 21.4% in the probiotic lozenge group. Goyal *et al*. [19] reported that the use of probiotic mouthwashes significantly decreased the levels of *Porphyromonas gingivalis* by 1.6 × 10^6^ CFU/ml (*P* < .05). Conversely, Habib [31] assessed the levels of other periodontal pathogens (*P. intermedia*, *C. rectus*, and *F. nucleatum*) in the subgingival plaque and found no significant changes (*P* > .05).

### Meta-analysis

**Figure 3.**
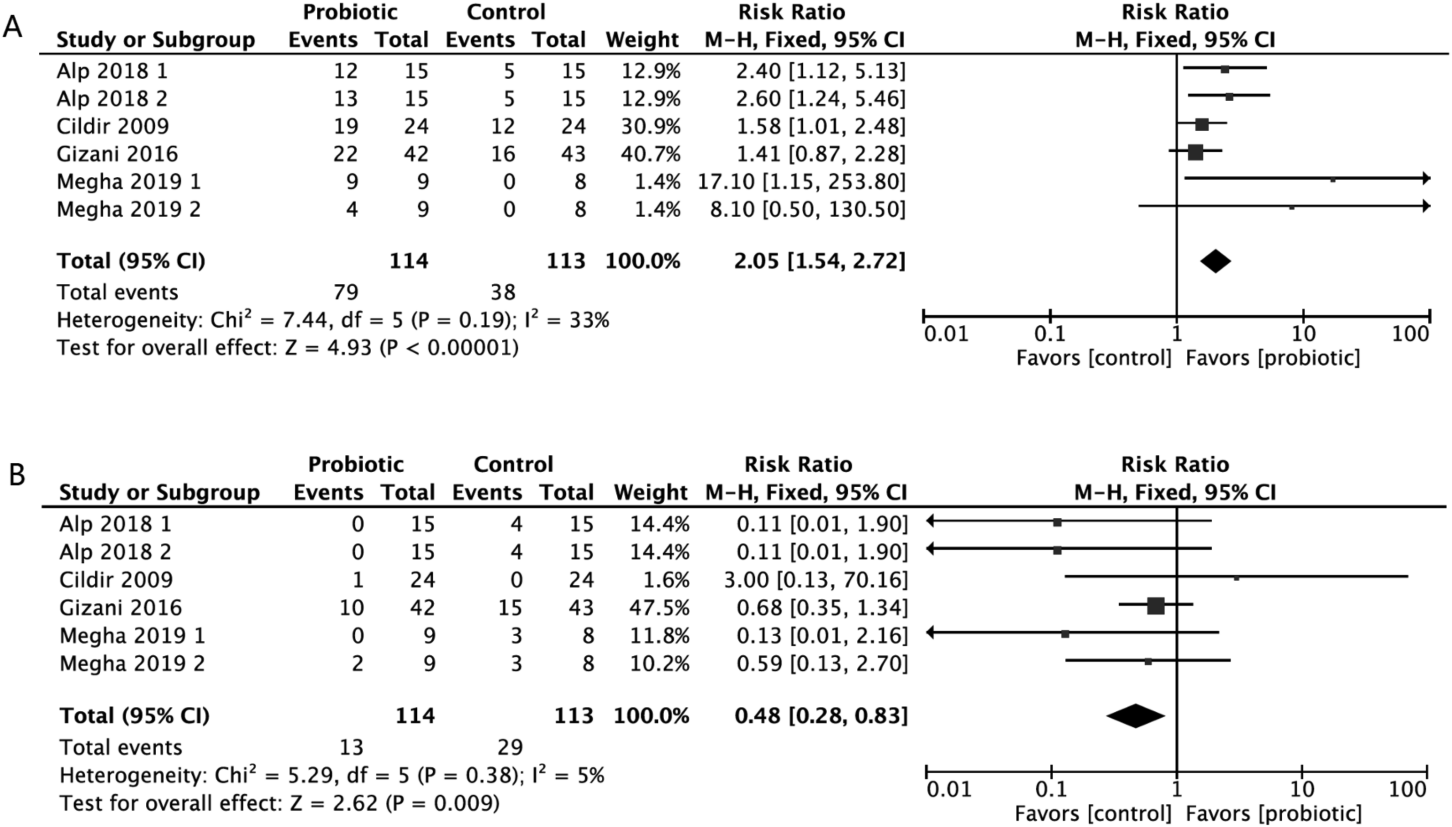
Forest plots depicting the comparison between the probiotic and control groups after treatment: (a) *S. mutans* <10^5^ CFU/ml; (b) *S. mutans* >10^6^ CFU/ml.

**Figure 4.**
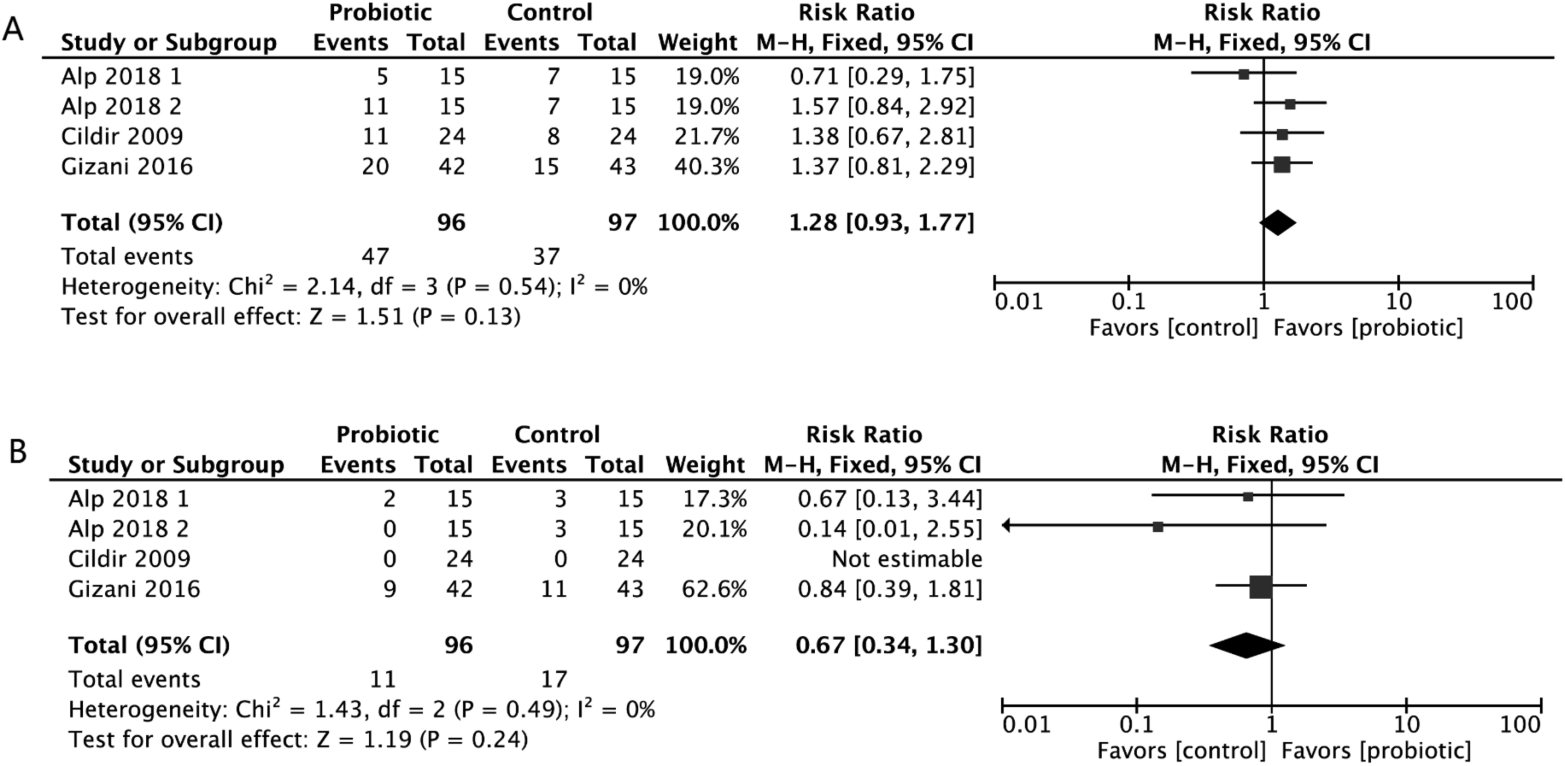
Forest plots depicting the comparison between the probiotic and control groups after treatment: (a) *Lactobacillus* <10^5^ CFU/ml; (b) *Lactobacillus* >10^6^ CFU/ml.

Of the 15 studies, three studies classified as having a ‘high RoB’ were excluded from the meta-analysis [19, 21, 29]. Finally, four studies [22, 32, 34, 36] were included in a dichotomous meta-analysis as information on the proportion of patients with low, medium, or high counts of salivary *S. mutans* or *Lactobacillus* could be extracted. These RCTs used a chair-side test for evaluating the levels of *S. mutans* or *Lactobacillus*. In the first meta-analysis for low *S.mutans* counts (<10^5^ CFU/ml), the results showed that 79 (69.3%) of the 114 individuals who used probiotics had low *S.mutans* counts (<10^5^ CFU/ml), compared to 38 (33.6%) of the 113 participants in the placebo group. This finding indicates that probiotics significantly increased the likelihood of reducing the abundance of *S. mutans* to <10^5^ CFU/ml (RR: 2.05 [1.54, 2.72]; *P* < .001; *I*^2^ = 33%) (Fig. 3a). In the second meta-analysis for high *S.mutans* counts (>10^6^ CFU/ml), 13 (11.4%) of the 114 individuals in the probiotic group exhibited high *S.mutans* counts (>10^6^ CFU/ml), compared to 29 (25.7%) of the 113 patients in the control group. The supplementation of probiotics significantly reduced the likelihood of increasing this abundance to >10^6^ CFU/ml (RR: 0.48 [0.28, 0.83]; *P* = .009; *I*^2^ = 5%) (Fig. 3b). In the third and fourth meta-analyses for low and high *Lactobacillus* counts, patients that used (*n* = 47) and did not use (*n* = 37) probiotics exhibited low *Lactobacillus* counts (<10^5^ CFU/ml), and 11 patients in the probiotic group and 17 patients in the control group showed high *Lactobacillus* counts (>10^6^ CFU/ml). No significant differences were observed in the abundance of *Lactobacillus* at either <10^5^ CFU/ml (RR: 1.28 [0.93, 1.77]; *P* = .13; *I*^2^ = 0%) (Fig. 4a) or >10^6^ CFU/ml (RR: 0.67 [0.34,1.30]; *P* = .24; *I*^2^ = 0%) (Fig. 4b). Quantitative analyses for other outcome parameters could not be conducted due to insufficient data and the diverse measuring methods. The quality of evidence on the microbial measures according to the GRADE approach was moderate due to limited sample sizes (Supplementary Table 2).

## Discussion

Probiotics suppress the growth of pathogenic microorganisms by producing antimicrobial compounds and competing for adhesion sites with pathogens [11], offering an alternative for the prevention and treatment of caries and gingivitis in patients undergoing orthodontic treatment [23, 24]. This systematic review and meta-analysis aimed to summarise the recently published data and evaluate whether supplementation with probiotics is beneficial to the oral health of patients undergoing orthodontic treatment. The outcome measurements include periodontal indexes, incidence of WSLs, and bacterial counts, which are the most commonly affected oral health-related measures during orthodontic treatment.

In general, the clinical effectiveness of probiotics for orthodontic patients is conflicting. Four studies reported no significant changes in PI in the probiotic group [20, 26, 29, 30], and two demonstrated that probiotics did not significantly reduce GI [20, 31] However, Shah *et al*. [21] reported that the probiotic group’s build-up of plaque and gingival inflammation were significantly decreased. One study assessing the WSLs reported that there was no significant difference in the incidence of WSLs between the probiotic and placebo groups at debonding [32]. The variations in the probiotic administration vehicle, concentration, strains, and intervention duration could explain these controversial results.

Concerning the administration methods, probiotics were found to be positive in most studies when used in mouth rinses and dairy products, including yoghurt and curd [19, 21, 22, 27, 35, 36]. Dairy products such as milk and yoghurt are generally regarded as effective probiotic administration vehicles. These products include calcium phosphate, ammonia, and casein, improving enamel remineralisation, raising plaque-PH, and preventing bacterial cells from adhering to the teeth [37]. According to a systematic review and meta-analysis, the dairy products used in 14 of the 20 trials substantially reduced the *S. mutans* levels [38]. The use of mouth rinses is efficient since these can reach the tooth surface easily, including around the brackets and archwires, leading to the effective colonisation of the probiotic microorganisms in the oral cavity [21]. By contrast, six studies that demonstrated no positive effects of probiotics used lozenges as the probiotic carrier, suggesting lozenges may not be effective vehicles in promoting oral health [20, 26, 29–32].

The optimal strains and concentrations of probiotics for use in oral health maintenance are yet to be clarified. A large number of current dental research articles used probiotic doses ranging from 10^8^ to 10^9^ CFU [32, 39, 40]. Most of the studies included in this systematic review used probiotic doses within this range, however, in one study, Ebrahim *et al*. used a concentration of 10^5^ CFUs/lozenge and failed to observe any clinically detectable effects on plaque build-up [26]. The authors considered that the lack of significant changes could be due to the low concentration of probiotics. Apart from variations in concentrations, there was also a diversity of probiotic bacterial species. *Lactobacillus* and *Bifidobacterium* species are the most common probiotic microorganisms used for oral health, with most of the studies in this review using these two species [19, 21, 22, 25–27, 29–33, 35, 36]. Similar to probiotics used in the gastrointestinal system, a combination of probiotic bacterial strains may exert a synergistic effect against oral disorders [41]. Alp and Baka, Goyal *et al*., and Megha *et al*. used a mixture of probiotic bacterial strains and observed a significant decrease in the pathogen levels [19, 22, 34]. By contrast, the Lorodent probiotic complex, which contains five *Lactobacillus* strains, exhibited no positive effects in reducing plaque accumulation and *S. mutans* counts in patients undergoing orthodontic treatment [26, 30, 31]. It is crucial to consider the unique orthodontic environment when determining the optimal probiotic concentration and strain for patients undergoing orthodontic treatment. Oral biofilms in these individuals tend to be thicker and more pathogenic compared to those in the general population, primarily due to the adverse effects of fixed orthodontic appliances on oral hygiene [42]. Previous research has revealed that the *S. mutans* counts were four times higher in patients undergoing orthodontic treatment than in the general population [43]. Therefore, future studies are still required to determine the optimal species and concentrations of probiotics to be used as a supplement for orthodontic patients.

In terms of the microbiological outcomes, five studies [21, 22, 34–36] concluded that probiotic use can significantly reduce *S. mutans* counts in the saliva or plaque of patients, whereas the other six studies reported contradictory results [25–27, 30, 32, 33]. Alp and Baka [34] and Gizani *et al*. [32] found significant decreases in the *Lactobacillus* counts in the saliva of patients in the probiotic group, whereas the other four RCTs showed no considerable alternations [27, 30, 33, 36]. The possible reasons for the contradictory microbiological outcomes could be the clinical heterogeneity factors mentioned before. An additional reason is the different methods used to assess the microorganism counts. There are three methods for assessing microorganism counts: traditional plate counting, real-time quantitative polymerase chain reaction, and chair-side test kits. The chair-side tests correspond well with traditional laboratory methods, are simple to handle in clinical settings, and are thus extensively used in evaluating bacterial levels [32, 36, 44]. The four studies included in the meta-analysis used the chair-side test for evaluating the levels of *S. mutans* or *Lactobacillus* [22, 32, 34, 36]. A meta-analysis was conducted on these four studies that summarised the number of patients with different levels of *S. mutans* or *Lactobacillus* before and after probiotic use. The results showed that when the probiotic and control groups were compared after treatment, the probiotic group had more patients with low salivary *S. mutans* counts (<10^5^ CFU/ml) and fewer patients with high counts (>10^6^ CFU/ml); however, no such significant effects were observed for *Lactobacillus*. The results suggested that probiotics significantly increased the likelihood of reducing the abundance of *S. mutans* to below 10^5^ CFU/ml and reduced the likelihood of increasing the abundance of *S. mutans* to beyond 10^6^ CFU/ml. This result corroborates those of other meta-analyses conducted in the general population without orthodontic treatment: three meta-analyses concluded that probiotic therapy in the general population may decrease the *S. mutans* counts but have no effects on *Lactobacillus* [38, 45, 46]. Based on these results, probiotics might have a potential preventive effect on dental caries by reducing the *S. mutans* counts in saliva. However, whether this reduction in *S. mutans* counts in orthodontic patients has any clinical significance remains to be elucidated. In addition, *Lactobacillus* counts were not significantly changed when comparing the probiotic and control groups at the end of the intervention. The reason underlying the nonsignificant differences in the *Lactobacillus* counts may be explained by the fact that some of the studies used *Lactobacillus* as the probiotic strain, which may have masked the ultimate bacterial counts [38].

It is worth noting that although the probiotic strains varied in the current analysis, previous research suggested that some observed probiotic effects are traits shared by multiple probiotic species rather than just a few well-studied strains [47, 48]. For instance, Caglar and colleagues conducted several studies examining the change in saliva *S. mutans* counts after the consumption of various probiotics, including *Bifidobacterium lactis* Bb-12, *Lactobacillus reuteri* ATCC 55730 and ATCC PTA 5289, *Bifidobacterium* DN-173010, using different delivery methods (chewing gum, yoghurt, water, and tablets) [49–51]. The results demonstrated a significant decrease in *S. mutans* counts in all studies, independent of the probiotic strain used. Therefore, the current study focuses on the overall effects rather than differentiating between specific probiotic species, thereby encompassing a broader understanding of the collective impact of probiotics in orthodontic treatment.

### Limitations

Despite a comprehensive search strategy, there is a shortage of high-quality RCTs with adequate sample size to make clinical recommendations. Current studies lack standardisation in their probiotic protocols, resulting in variations in strains, concentrations, intervention durations, and follow-up durations. Additionally, subgroup or correlation analyses were not possible due to the heterogeneities between the studies and the limited sample sizes. Owing to these methodological shortcomings, the conclusions should be generalised with caution.

## Conclusion

There is insufficient evidence to determine the clinical benefits of probiotics on the oral health of patients undergoing orthodontic treatment. Probiotics may have potential benefits in reducing salivary *S. mutans* counts; however, whether this reduction in *S. mutans* counts has any clinical significance remains to be elucidated. Future studies should be conducted to determine the optimum delivery system, appropriate probiotic strains, and effective concentrations and should have a longer follow-up duration. Moreover, the effects of probiotics on the oral health of patients undergoing orthodontic treatment with other appliances, such as clear aligners and lingual appliances, should also be explored.

## Supplementary Material

cjad046_suppl_Supplementary_Table_S1

cjad046_suppl_Supplementary_Table_S2

## Data Availability

The data underlying this article will be shared on reasonable request to the corresponding author.

## References

[1] Guo R , Lin Y, Zheng Y et al. The microbial changes in subgingival plaques of orthodontic patients: a systematic review and meta-analysis of clinical trials. BMC Oral Health 2017;17:90. 10.1186/s12903-017-0378-1 28576147 PMC5455174

[2] Mummolo S , Nota A, Albani F et al. Salivary levels of *Streptococcus mutans* and Lactobacilli and other salivary indices in patients wearing clear aligners versus fixed orthodontic appliances: an observational study. PLoS One 2020;15:e0228798. 10.1371/journal.pone.0228798 32330172 PMC7182227

[3] Tufekci E , Dixon JS, Gunsolley JC et al. Prevalence of white spot lesions during orthodontic treatment with fixed appliances. Angle Orthod 2011;81:206–10. 10.2319/051710-262.1 21208070 PMC8925248

[4] Boke F , Gazioglu C, Akkaya S et al. Relationship between orthodontic treatment and gingival health: a retrospective study. Eur J Dent 2014;8:373–80. 10.4103/1305-7456.137651 25202219 PMC4144137

[5] Lucchese A , Gherlone E. Prevalence of white-spot lesions before and during orthodontic treatment with fixed appliances. Eur J Orthod 2013;35:664–8. 10.1093/ejo/cjs070 23045306

[6] Davis SM , Plonka A, Fulks BA et al. Consequences of orthodontic treatment on periodontal health: clinical and microbial effects. Semin Orthod 2014;20:139–49.

[7] Low B , Lee W, Seneviratne CJ et al. Ultrastructure and morphology of biofilms on thermoplastic orthodontic appliances in ‘fast’ and ‘slow’ plaque formers. Eur J Orthod 2011;33:577–83. 10.1093/ejo/cjq126 21187528

[8] Tasios T , Papageorgiou SN, Papadopoulos MA et al. Prevention of orthodontic enamel demineralization: a systematic review with meta-analyses. Orthod Craniofac Res 2019;22:225–35. 10.1111/ocr.12322 31081584

[9] Niazi FH , Kamran MA, Naseem M et al. Anti-plaque efficacy of herbal mouthwashes compared to synthetic mouthwashes in patients undergoing orthodontic treatment: a randomised controlled trial. Oral Health Prev Dent 2018;16:409–16. 10.3290/j.ohpd.a40983 30151504

[10] Morelli L , Capurso L. FAO/WHO guidelines on probiotics: 10 years later. J Clin Gastroenterol 2012;46:S1–2. 10.1097/mcg.0b013e318269fdd5 22955349

[11] Teughels W , Van Essche M, Sliepen I et al. Probiotics and oral healthcare. Periodontology 2000 2008;48:111–47. 10.1111/j.1600-0757.2008.00254.x 18715361

[12] Santosa S , Farnworth E, Jones PJ. Probiotics and their potential health claims. Nutr Rev 2006;64:265–74. 10.1111/j.1753-4887.2006.tb00209.x 16808112

[13] Twetman S , Keller MK. Probiotics for caries prevention and control. Adv Dent Res 2012;24:98–102. 22899689 10.1177/0022034512449465

[14] Yanine N , Araya I, Brignardello-Petersen R et al. Effects of probiotics in periodontal diseases: a systematic review. Clin Oral Investig 2013;17:1627–34. 10.1007/s00784-013-0990-7 23657745

[15] López-Valverde N , López-Valverde A, Macedo De Sousa B et al. Role of probiotics in halitosis of oral origin: a systematic review and meta-analysis of randomized clinical studies. Front Nutr 2021;8:787908. 10.3389/fnut.2021.787908 35127785 PMC8813778

[16] Pahumunto N , Piwat S, Chankanka O et al. Reducing mutans streptococci and caries development by *Lactobacillus paracasei* SD1 in preschool children: a randomized placebo-controlled trial. Acta Odontol Scand 2018;76:331–7. 10.1080/00016357.2018.1453083 29566582

[17] Vicario M , Santos A, Violant D et al. Clinical changes in periodontal subjects with the probiotic *Lactobacillus reuteri* Prodentis: a preliminary randomized clinical trial. Acta Odontol Scand 2013;71:813–9. 10.3109/00016357.2012.734404 23176716

[18] Lee DS , Lee SA, Kim M et al. Reduction of halitosis by a tablet containing *Weissella cibaria* CMU: a randomized, double-blind, placebo-controlled study. J Med Food 2020;23:649–57. 10.1089/jmf.2019.4603 32379992

[19] Goyal N , Shamanna PU, Varughese ST et al. Effects of amine fluoride and probiotic mouthwash on levels of *Porphyromonas gingivalis* in orthodontic patients: a randomized controlled trial. J Indian Soc Periodontol 2019;23:339–44. 10.4103/jisp.jisp_551_18 31367131 PMC6628766

[20] Benic GZ , Farella M, Morgan XC et al. Oral probiotics reduce halitosis in patients wearing orthodontic braces: a randomized, triple-blind, placebo-controlled trial. J Breath Res 2019;13:036010. 10.1088/1752-7163/ab1c81 31022704

[21] Shah SS , Nambiar S, Kamath D et al. Comparative evaluation of plaque inhibitory and antimicrobial efficacy of probiotic and chlorhexidine oral rinses in orthodontic patients: a randomized clinical trial. Int J Dent 2019;2019:1964158. 10.1155/2019/1964158 30930947 PMC6410424

[22] Megha S , Shalini G, Varsha SA et al. Effect of short-term placebo-controlled consumption of probiotic yoghurt and Indian curd on the streptococcus mutans level in children undergoing fixed interceptive orthodontic therapy. Turk J Orthod 2019;32:16–21. 10.5152/TurkJOrthod.2019.18016 30944895 PMC6436904

[23] Hadj-Hamou R , Senok AC, Athanasiou AE et al. Do probiotics promote oral health during orthodontic treatment with fixed appliances? A systematic review. BMC Oral Health 2020;20:126. 10.1186/s12903-020-01109-3 32334590 PMC7183645

[24] Pietri FK , Rossouw PE, Javed F et al. Role of probiotics in oral health maintenance among patients undergoing fixed orthodontic therapy: a systematic review of randomized controlled clinical trials. Probiotics Antimicrob Proteins 2020;12:1349–59. 10.1007/s12602-020-09683-2 32623645

[25] Dadgar S , Heydarian A, Sobouti F et al. Effects of probiotic and fluoride mouthrinses on *Streptococcus mutans* in dental plaque around orthodontic brackets: a preliminary explorative randomized placebo-controlled clinical trial. Dent Res J 2021;18:74. 10.4103/1735-3327.326647 PMC8543094 34760065

[26] Ebrahim F , Malek S, James K et al. Effectiveness of the lorodent probiotic lozenge in reducing plaque and *Streptococcus mutans* levels in orthodontic patients: a double-blind randomized control trial. Front Oral Health 2022;3:884683. 35571981 10.3389/froh.2022.884683 PMC9093136

[27] Alforaidi S , Bresin A, Almosa N et al. Effect of drops containing *Lactobacillus reuteri* (DSM 17938 and ATCC PTA 5289) on plaque acidogenicity and other caries-related variables in orthodontic patients. BMC Microbiol 2021;21:271. 10.1186/s12866-021-02310-2 34615458 PMC8496079

[28] Guyatt GH , Oxman AD, Vist GE et al.; GRADE Working Group. GRADE: an emerging consensus on rating quality of evidence and strength of recommendations. BMJ 2008;336:924–6. 10.1136/bmj.39489.470347.AD 18436948 PMC2335261

[29] Kohar NM , Emmanuel V, Astuti L. Comparison between probiotic lozenges and drinks towards periodontal status improvement of orthodontic patients. Dent J (Majalah Kedokteran Gigi) 2015;48:126–9.

[30] Jivraj FE. Effectiveness of the Lorodent Probiotic Lozenge in Reducing Plaque and S. Mutans Levels in Orthodontic Patients: a Randomized, Double-Blind, Placebo-Controlled Trial. Canada: University of Toronto, 2015.

[31] Habib S. Assessment of the Therapeutic Potential of a Dental Probiotic in Orthodontic Patients Affected by Gingivitis: A Randomized Control Trial. Canada: University of Toronto, 2016.

[32] Gizani S , Petsi G, Twetman S et al. Effect of the probiotic bacterium *Lactobacillus reuteri* on white spot lesion development in orthodontic patients. Eur J Orthod 2016;38:85–9. 10.1093/ejo/cjv015 25840585

[33] Pinto GS , Cenci MS, Azevedo MS et al. Effect of yogurt containing *Bifidobacterium animalis* subsp. lactis DN-173010 probiotic on dental plaque and saliva in orthodontic patients. Caries Res 2014;48:63–8. 10.1159/000353467 24217196

[34] Alp S , Baka ZM. Effects of probiotics on salivary *Streptecoccus mutans* and *Lactobacillus* levels in orthodontic patients. Am J Orthod Dentofacial Orthop 2018;154:517–23. 30268262 10.1016/j.ajodo.2018.01.010

[35] Jose JE , Padmanabhan S, Chitharanjan AB. Systemic consumption of probiotic curd and use of probiotic toothpaste to reduce *Streptococcus mutans* in plaque around orthodontic brackets. Am J Orthod Dentofacial Orthop 2013;144:67–72. 10.1016/j.ajodo.2013.02.023 23810047

[36] Cildir SK , Germec D, Sandalli N et al. Reduction of salivary mutans streptococci in orthodontic patients during daily consumption of yoghurt containing probiotic bacteria. Eur J Orthod 2009;31:407–11. 10.1093/ejo/cjn108 19193706

[37] Kargul B , Caglar E, Lussi A. Erosive and buffering capacities of yogurt. Quintessence Int 2007;38:381–5. 17568836

[38] Nadelman P , Magno MB, Masterson D et al. Are dairy products containing probiotics beneficial for oral health? A systematic review and meta-analysis. Clin Oral Investig 2018;22:2763–85. 10.1007/s00784-018-2682-9 30298454

[39] Näse L , Hatakka K, Savilahti E et al. Effect of long-term consumption of a probiotic bacterium, *Lactobacillus rhamnosus* GG, in milk on dental caries and caries risk in children. Caries Res 2001;35:412–20. 10.1159/000047484 11799281

[40] Stecksén-Blicks C , Sjöström I, Twetman S. Effect of long-term consumption of milk supplemented with probiotic lactobacilli and fluoride on dental caries and general health in preschool children: a cluster-randomized study. Caries Res 2009;43:374–81. 10.1159/000235581 19690413

[41] Timmerman HM , Koning CJ, Mulder L et al. Monostrain, multistrain and multispecies probiotics—A comparison of functionality and efficacy. Int J Food Microbiol 2004;96:219–33. 10.1016/j.ijfoodmicro.2004.05.012 15454313

[42] Sudjalim TR , Woods MG, Manton DJ et al. Prevention of demineralization around orthodontic brackets in vitro. Am J Orthod Dentofacial Orthop 2007;131:705.e1–9. 10.1016/j.ajodo.2006.09.043 17561043

[43] Rosenbloom RG , Tinanoff N. Salivary *Streptococcus mutans* levels in patients before, during, and after orthodontic treatment. Am J Orthod Dentofacial Orthop 1991;100:35–7. 10.1016/0889-5406(91)70046-Y 2069145

[44] Tanabe Y , Park JH, Tinanoff N et al. Comparison of chairside microbiological screening systems and conventional selective media in children with and without visible dental caries. Pediatr Dent 2006;28:363–8. 16903447

[45] Laleman I , Detailleur V, Slot DE et al. Probiotics reduce mutans streptococci counts in humans: a systematic review and meta-analysis. Clin Oral Investig 2014;18:1539–52. 10.1007/s00784-014-1228-z 24663813

[46] Gruner D , Paris S, Schwendicke F. Probiotics for managing caries and periodontitis: Systematic review and meta-analysis. J Dent 2016;48:16–25. 10.1016/j.jdent.2016.03.002 26965080

[47] Haukioja A , Yli-Knuuttila H, Loimaranta V et al. Oral adhesion and survival of probiotic and other lactobacilli and bifidobacteria in vitro. Oral Microbiol Immunol 2006;21:326–32. 10.1111/j.1399-302X.2006.00299.x 16922933

[48] Cosseau C , Devine DA, Dullaghan E et al. The commensal *Streptococcus salivarius* K12 downregulates the innate immune responses of human epithelial cells and promotes host-microbe homeostasis. Infect Immun 2008;76:4163–75. 10.1128/IAI.00188-08 18625732 PMC2519405

[49] Caglar E , Kuscu OO, Selvi Kuvvetli S et al. Short-term effect of ice-cream containing *Bifidobacterium lactis* Bb-12 on the number of salivary mutans streptococci and lactobacilli. Acta Odontol Scand 2008;66:154–8. 10.1080/00016350802089467 18568474

[50] Caglar E , Kavaloglu SC, Kuscu OO et al. Effect of chewing gums containing xylitol or probiotic bacteria on salivary mutans streptococci and lactobacilli. Clin Oral Investig 2007;11:425–9. 10.1007/s00784-007-0129-9 17574481

[51] Caglar E , Cildir SK, Ergeneli S et al. Salivary mutans streptococci and lactobacilli levels after ingestion of the probiotic bacterium *Lactobacillus reuteri* ATCC 55730 by straws or tablets. Acta Odontol Scand 2006;64:314–8. 10.1080/00016350600801709 16945898

