# Supplementary material for: Effects of probiotics on the oral health of patients undergoing orthodontic treatment: a systematic review and meta-analysis
Source: Eur J Orthod. 2023 Aug 8;45(5):599–611. doi: 10.1093/ejo/cjad046 (PMC10505686; doi:10.1093/ejo/cjad046)
Supplement: cjad046_suppl_Supplementary_Table_S2 [file cjad046_suppl_supplementary_table_s2.pdf]

Supplementary Table 2. A summary of the quality of the evidence from the meta-analyses using the Grading of Recommendations Assessment, Development and Evaluation (GRADE)

Certainty assessment							№ of patients		Effect		Certainty
№ of studies	Study design	Risk of bias	Inconsistency	Indirectness	Imprecision	Other considerations	Probiotic	Control	Relative (95% CI)	Absolute (95% CI)	
**S. mutans < 10^5 CFU/ml**											
6	randomised trials	not serious	not serious	not serious	serious[a]	none	79/114 (69.3%)	38/113 (33.6%)	**RR 2.05** (1.54 to 2.72)	**353 more per 1,000** (from 182 more to 578 more)	⊕⊕⊕◯ Moderate
**S. mutans > 10^6 CFU/ml**											
6	randomised trials	not serious	not serious	not serious	serious[a]	none	13/114 (11.4%)	29/113 (25.7%)	**RR 0.48** (0.28 to 0.83)	**133 fewer per 1,000** (from 185 fewer to 44 fewer)	⊕⊕⊕◯ Moderate
**Lactobacillus <10^5 CFU/ml**											
4	randomised trials	not serious	not serious	not serious	serious[a]	none	47/96 (49.0%)	37/97 (38.1%)	**RR 1.28** (0.93 to 1.77)	**107 more per 1,000** (from 27 fewer to 294 more)	⊕⊕⊕◯ Moderate
**Lactobacillus >10^6 CFU/ml**											
4	randomised trials	not serious	not serious	not serious	serious[a]	none	11/96 (11.5%)	17/97 (17.5%)	**RR 0.67** (0.34 to 1.30)	**58 fewer per 1,000** (from 116 fewer to 53 more)	⊕⊕⊕◯ Moderate

CI: confidence interval; **RR:** risk ratio

Explanations: a. small sample size
